# Hemorragia postparto: intervenciones y tratamiento del profesional de enfermería para prevenir shock hipovolémico**

**DOI:** 10.15649/cuidarte.2075

**Published:** 2022-08-14

**Authors:** Ruth Alexandra Castiblanco Montañez, Cyndi Mileni Coronado Veloza, Laura Valentina Morales Ballesteros, Tatiana Valentina Polo González, Angie Julieth Saavedra Leyva

**Affiliations:** 1 Fundación Universitaria de Ciencias de la Salud-FUCS. Bogotá- Colombia. Email: racastiblanco@ fusalud.edu.co Fundación Universitaria de Ciencias de la Salud Bogotá Colombia racastiblanco@ fusalud.edu.co; 2 Fundación Universitaria de Ciencias de la Salud-FUCS. Bogotá- Colombia. Email: cmcoronado@fucsalud.edu.co Fundación Universitaria de Ciencias de la Salud Bogotá Colombia cmcoronado@fucsalud.edu.co; 3 Fundación Universitaria de Ciencias de la Salud-FUCS. Bogotá- Colombia. Email: lvmorales@fucsalud.edu.co Fundación Universitaria de Ciencias de la Salud Bogotá Colombia lvmorales@fucsalud.edu.co; 4 Fundación Universitaria de Ciencias de la Salud-FUCS. Bogotá- Colombia. Email: tvpolo@fucsalud.edu.co Fundación Universitaria de Ciencias de la Salud Bogotá Colombia tvpolo@fucsalud.edu.co; 5 Fundación Universitaria de Ciencias de la Salud-FUCS. Bogotá-Colombia. Email: ajsaavedra@fucsalud.edu.co Fundación Universitaria de Ciencias de la Salud-FUCS Bogotá Colombia ajsaavedra@fucsalud.edu.co

**Keywords:** Hemorragia Posparto, Cuidado de Enfermería, Shock hipovolémico, Postpartum haemorrhage, Nursing Care, Hypovolemic Shock, Hemorragia Pós-Parto, Cuidados de Enfermagem, Choque hipovolêmico

## Abstract

**Introducción:**

En Colombia la hemorragia postparto es la segunda causa de mortalidad en mujeres gestantes de 24 a 34 años con 6,9 casos por cada 1000 nacidos vivos. Después del parto se prevé que el 8.2% de las mujeres latinoamericanas presentarán hemorragia postparto.

**Objetivo:**

Describir el cuidado de enfermería a mujeres que presentan hemorragia postparto para disminuir el riesgo de shock hipovolémico, a través de una revisión integrativa de la literatura.

**Metodología:**

Revisión integrativa de la literatura siguiendo la propuesta por Sasso, de Campos y Galvão, se realizó una búsqueda en ClinicalKey, LILACS, CINAHL, Epistemonikos, Cochrane Library, PubMed, Scielo y Google Scholar; se incluyeron artículos publicados en los últimos cinco años, en español, inglés y portugués, se clasificaron por nivel de evidencia y grado de recomendación. Esta investigación es de bajo riesgo por ser de tipo documental.

**Resultados:**

Se recopilaron 41 artículos definitivos. La información se organizó en: cuadro clínico, cuidados de enfermería y dificultades en la atención gineco-obstétrica.

**Discusión:**

El profesional de enfermería debe identificar barreras en la atención evaluando la capacidad resolutiva de las instituciones y analizando los casos de muerte materna. Se recomienda el uso de misoprostol con oxitocina o únicamente de carbetocina y la combinación de ergometrina con oxitocina según el volumen de sangrado.

**Conclusión:**

Es pertinente realizar un examen físico para reconocer signos de inestabilidad hemodinámica, y de shock hipovolémico. Además, los diagnósticos e intervenciones de enfermería se enfocan en brindar cuidados de calidad, para evitar complicaciones como la muerte.

## Abstract (en)

**Introduction:**

In Colombia, postpartum hemorrhage is the second leading cause of death in pregnant women at a rate of 6.9 deaths per 1,000 live births. After childbirth, 8.2% of Latin Amer- ican women are expected to have postpartum hemorrhage.

**Objective:**

To describe nursing care delivered to women with postpartum hemorrhage to reduce the risk of hypovolemic shock by means of an integrative literature review.

**Materials and Methods:**

Following the approach suggested by Sasso, de Campos and Galvão, an integrative literature review was conducted on ClinicalKey, LILACS, CINAHL, Epistemonikos, Cochrane Library, PubMed and ScieELO Google Scholar databases. Articles published in the last five years in Spanish, English and Portuguese were selected and classified by their level of evidence and degree of recommendation. This is a low-risk research due to its documentary nature.

**Results:**

41 articles were finally selected and classified into clinical picture, nursing care and challenges in OBGYN care.

**Discussion:**

Nursing professionals should identify barriers to care by evaluating institutional problem-solving ca- pacity and analyzing maternal death cases. Using misoprostol and oxytocin or only carbetocin and ergometrine-oxytocin is suggested based on the quantity of blood loss.

**Conclusions:**

Per- forming a physical examination is important to recognize signs of hemodynamic instability and hypovolemic shock. Nursing diagnoses and interventions focus on providing quality patient care to prevent certain complications such as death.

## Resumo

**Introdução:**

Na Colômbia, a hemorragia pós-parto é a segunda principal causa de mortalidade em mulheres grávidas de 24-34 anos, com 6,9 casos por 1000 nascidos vivos. Após o parto, 8,2% das mulheres latino-americanas devem sofrer de hemorragia pós-parto.

**Objetivo:**

Descrever os cuidados de enfermagem a mulheres com hemorragia pós-parto para reduzir o risco de choque hipovolêmico, através de uma revisão integrativa da bibliografia.

**Metodologia:**

Revisão inte- grativa da bibliografia seguindo a proposta de Sasso, de Campos e Galvão, foi realizada uma pesquisa em ClinicalKey, LILACS, CINAHL, Epistemonikos, Cochrane Library, PubMed, Scielo e Google Scholar; foram incluídos artigos publicados nos últimos cinco anos em espanhol, inglês e português, classificados por nível de evidência e grau de recomendação. Esta é uma pesquisa de baixo risco, pois é do tipo documental.

**Resultados:**

Foram recolhidos 41 artigos definitivos. A informação foi organizada em: quadro clínico, cuidados de enfermagem e dificuldades nos cuidados gineco-obstetrítricos.

**Discussão:**

O profissional de enfermagem deve identificar as barreiras ao cuidado, avaliando a capacidade das instituições e analisando os casos de morte materna. O uso de misoprostol com ocitocina, ou carbetocina isoladamente, e a combinação de ergometrina com ocitocina são recomendados dependendo do volume de sangramento.

**Conclusão:**

O exame físico para sinais de instabilidade hemodinâmica e choque hipovolêmico é relevante. Além disso, os diagnósticos e intervenções de enfermagem concentram-se na pres- tação de cuidados de qualidade para evitar complicações como a morte.

## Introducción

El embarazo es el resultado de la implantación del cigoto en el útero hasta el momento del parto, éste consta de fase de dilatación, período expulsivo, y período de alumbramiento; después del mismo se pueden desarrollar complicaciones como: hipertensión gestacional; infecciones y hemorragia postparto (HPP), siendo esta la que se presenta con mayor prevalencia, lo que podría desencadenar shock hipovolémico, de acuerdo al grado de pérdida sanguínea, la rapidez de la separación de la placenta y la efectividad de la contracción uterina^1^, ^2^.

La HPP se clasifica en temprana si se presenta durante las primeras 24 horas, generalmente en las dos primeras horas, y tardía si ocurre entre las 24 y las 6 semanas del posparto, y se caracteriza por una pérdida estimada >500 ml de sangre; pérdida de todo el volumen sanguíneo en 24 horas; sangrado >150 ml/min; pérdida del 50% del volumen en 20 minutos y/o descenso del hematocrito ≥ 10%^3^. La HPP se presenta en el 5 al 15% de los partos y representa alrededor del 25-30% de muertes en gestantes <15 años, siendo en adolescentes la causa más frecuente de morbimortalidad a nivel mundial^4^, ^5^. Después del parto se prevé que el 8.2% de las mujeres latinoamericanas presentarán HPP grave y en Colombia es la segunda causa de mortalidad en mujeres gestantes, presentándose en edades de 24 a 34 años con 6,9 casos por cada 1000 nacidos vivos^6^, ^7^.

A nivel internacional se ha implementado la estrategia mundial para la salud de la mujer, el niño y el adolescente 2016-2030, en donde se propone la equidad en la atención en salud para poner fin a la mortalidad prevenible y brindar bienestar a dicha población, procurando que las mujeres puedan gozar con bienestar el embarazo y el parto^8^. Por otra parte, en Colombia se han realizado estudios con el fin de mediar la mortalidad materna realizando intervenciones apropiadas y oportunas durante el alumbramiento que han sido establecidas en las guías de código rojo^9^.

Sin embargo, aunque la HPP es prevenible en un 93%, no se detectan a tiempo los signos y síntomas en el puerperio. Por esta razón el profesional de enfermería debe actuar de manera ágil y eficaz durante el parto y en caso de hemorragia masiva para evitar complicaciones mayores^10^^,^^11^. Asimismo, a nivel social e institucional la HPP es percibida como: “una expresión de inequidad, desigualdad y falta de empoderamiento de las mujeres” puesto que según la OPS la HPP es una variable inherente de mortalidad en países de recursos bajos y medios, donde la atención en salud es deficiente; por lo que se considera importante la prestación de servicios en controles prenatales, partos y puerperio para prevenir la HPP^12^. Por esto, se pretende describir el cuidado de enfermería a mujeres que presentan hemorragia postparto para disminuir el riesgo de shock hipovolémico, a través de una revisión integrativa de la literatura.

## Materiales y Métodos

Investigación secundaria, con diseño de revisión integrativa de la literatura, siguiendo la propuesta por Sasso, de Campos y Galvão^13^^,^^14^, que sugiere seis fases para este proceso. En la primera, se estableció la pregunta PICO: ¿Cuál es el cuidado de enfermería a mujeres que presentan hemorragia post parto para disminuir el riesgo de shock hipovolémico? En la segunda fase, se formularon ecuaciones de búsqueda con los operadores booleanos AND y OR, utilizando los DeCS: Hemorragia posparto, Postpartum Hemorrhage, Hemorragia Pós- Parto. Nursing Care, Atención de enfermería, Cuidados de Enfermagem. Shock, Choque; y los MeSH: Immediate Postpartum Hemorrhage, Delayed Postpartum Hemorrhage. Nursing Care Management. Circulatory Failure, Circulatory Collapse, Hypovolemic shock.

Se rastrearon artículos en bases de datos como ClinicalKey, LILACS, CINAHL, Epistemonikos, Cochrane Library, en la interfaz PubMed, en el banco de artículos Scielo y en el meta buscador Google Scholar. La búsqueda se efectuó en marzo de 2020 teniendo en cuenta los siguientes criterios de selección: artículos publicados en los últimos cinco años, en idiomas español, inglés y portugués; se excluyeron los consensos de expertos e investigaciones que citaran mujeres post-cesárea y/o con patologías hematológicas y cardíacas como antecedentes.

En el desarrollo de la tercera fase, se revisó literatura gris a nivel nacional e internacional relacionada con guías de manejo propuestas para código rojo y las intervenciones desarrolladas por la Organización Mundial de la Salud (OMS) y la Organización Panamericana de la Salud (OPS). Posterior a esto, se construyó una matriz en Excel para registrar la búsqueda y extracción de unidades de análisis^15^, seleccionadas por título, resumen y texto completo, y clasificadas por nivel de evidencia y grado de recomendación según el Centro para la Medicina basado en la Evidencia (CBM)^16^.

**Figura 1:**
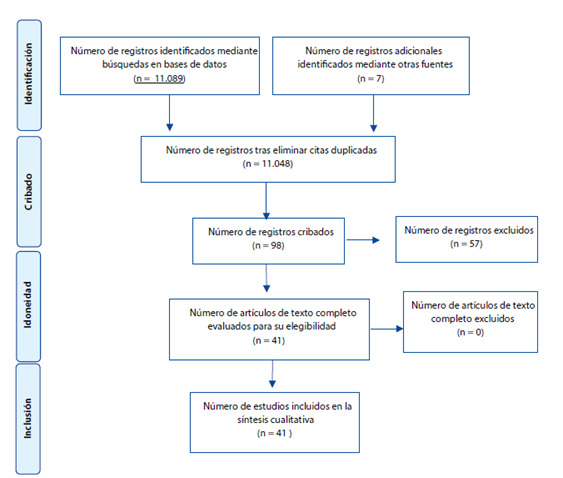
Diagrama de flujo de la búsqueda de literatura.

La búsqueda inicial arrojó 11.089 artículos de los cuales se descartaron 11.048, resultando 41 definitivos que reportan los principales cuidados de enfermería en la prevención de shock hipovolémico secundario a HPP. Estos artículos aportaron a la construcción de tres temáticas de la siguiente manera: Cuadro clínico (n=32), en donde se especifica la fisiopatología, factores, causas y diagnóstico de la HPP; La prevención, tratamiento e intervenciones de enfermería (n=35) y Dificultades que se presentan durante la atención gineco obstétrica (n=15). Cabe aclarar que algunas unidades contribuyeron a más de una categoría. Ver Figura 1.

Dentro de la cuarta fase se utilizaron instrumentos de valoración para la lectura crítica: para estudios de revisión sistemática CASPe^17^ con un puntaje 9 de 10 puntos, para estudios observacionales STROBE^18^ con un puntaje de 17 de 21 puntos, para ensayos clínicos aleatorizados CONSORT^19^ con un puntaje de 21 de 37 puntos y AGREE II^20^ para guías de práctica clínica.

Esta investigación cumplió con las consideraciones éticas contempladas en la Resolución 8430 de 1993 y se clasifica como investigación sin riesgo, puesto que no se realizan modificaciones en las variables de los individuos^21^, y con las disposiciones de la Ley 44 de 1993 correspondientes a los derechos de autor y a las consideraciones de la Ley 911 de 2004^22^^,^^23^.

## Resultados

Dentro de las unidades de análisis seleccionadas, se identificaron 17 en inglés (41,5%), 18 en español (43,9%) y 6 en portugués (14,6%), en su mayoría publicados en el año 2019 (36,5%). Con respecto al nivel de evidencia se encontró que el grado 3A (recomendación favorable) fue el más prevalente con un 41,46%, y de la misma forma se incluyeron 56% de investigaciones primarias. Ver Tabla 1

**Tabla 1:** Caracterización de las unidades de análisis.

Unidad	País	Idioma	Diseño	Nivel de evidencia	Grado de recomendación
Borovac^24^	Brasil	Inglés	Estudio de cohortes prospectivo	2B	Recomendación favorable
Escobar^25^	Colombia	Inglés	Estudio descriptivo de corte transversal, observacional	2C	Recomendación favorable
Okada^26^	Japón	Inglés	Estudio de cohortes retrospectivo	2B	Recomendación favorable
Sohn^27^	Sur de Corea	Inglés	Corte transversal	3B	Recomendación favorable
Pileggi^28^	Brasil	Inglés	Revisión sistemática de la literatura	2A	Recomendación favorable
Camacho^29^	Colombia	Inglés	Revisión sistemática	2A	Recomendación favorable
Durmaz^30^	Turquía	Inglés	Revisión sistemática - Meta-análisis	2A	Recomendación favorable
Feduniw^31^	Polonia	Inglés	Revisión sistemática	3A	Recomendación favorable
Corte^32^	Estados Unidos	Inglés	Revisión sistemática - Meta-análisis	1A	Extremadamente favorable
Crespo^33^	Ecuador	Español	Retrospectivo longitudinal, corte transversal	2C	Recomendación favorable
Hernández^34^	Chile	Español	Descriptivo de corte transversal	2C	Recomendación favorable
García^35^	México	Español	Revisión sistemática retrospectiva	3A	Recomendación favorable
López^36^	Colombia	Inglés	Descriptivo, serie de casos.	4	Recomendación favorable pero no concluyente
Tanaka^37^	Taiwán	Inglés	Revisión sistemática	3A	Recomendación favorable
Ferreira^38^	Brasil	Portugués	Revisión sistemática	3A	Recomendación favorable
Choque^39^	Perú	Español	Descriptivo, retrospectivo, corte transversal	2C	Recomendación favorable
Guía de cuidado de enfermería a la mujer en el periodo postparto^1^	Colombia	Español	Guía de práctica clínica	1C	Extremadamente favorable
Columbie^40^	Cuba	Español	Descriptivo, corte transversal, prospectivo	3A	Recomendación favorable
Paz^41^	Perú	Español	Corte transversal	2C	Recomendación favorable
Erazo^42^	Ecuador	Español	Descriptiva, serie de casos, explicativo	3A	Recomendación favorable
Rojas^43^	Colombia	Español	Descriptivo de corte transversal, analítico	2C	Recomendación favorable
Gámez^44^	México	Español	Caso clínico	3B	Recomendación favorable
Romero^45^	Colombia	Español	Corte transversal	2C	Recomendación favorable
Flores^46^	Ecuador	Español	Descriptico de corte transversal, retrospectivo	3A	Recomendación favorable
Tapia^47^	Ecuador	Español	Cohorte retrospectivo transversal	2A	Recomendación favorable
Lavaven^48^	Ecuador	Español	Revisión de literatura	3A	Recomendación favorable
Nagua^49^	Ecuador	Español	Descriptivo de análisis de caso	3A	Recomendación favorable
Oliva^50^	Perú	Español	Estudio de casos y controles	3A	Recomendación favorable
Suerda^51^	Brasil	Portugués	Revisión bibliográfica	3A	Recomendación favorable
De Siqueira^52^	Brasil	Portugués	Revisión sistemática de la literatura	3A	Recomendación favorable
Da Silva^53^	Brasil	Portugués	Revisión bibliográfica	3A	Recomendación favorable
Fortes^54^	Cabo Verde	Portugués	Revisión de la literatura	3A	Recomendación favorable
Koch^55^	Brasil	Inglés	Corte transversal	2C	Recomendación favorable
PPH CPG Work Group^56^	Canadá	Inglés	Guía de práctica clínica	1C	Extremadamente recomendable
Qayum^57^	Pakistán	Inglés	Ensayo clínico aleatorizado	1B	Extremadamente recomendable
King^58^	Sierra Leona	Inglés	Corte transversal	2A	Recomendación favorable
Kwon^59^	Corea	Inglés	Serie de casos	4	Recomendación favorable no concluyente
El-Garghy^60^	Egipto	Inglés	Estudio de cohortes	2B	Recomendación favorable
Treviño^61^	México	Español	Revisión sistemática	3A	Recomendación favorable
Teixeira^62^	Brasil	Portugués	Revisión sistemática	3A	Recomendación favorable
Morillas^63^	España	Español	Revisión bibliográfica	3A	Recomendación favorable

La información se organizó en tres temáticas: Cuadro clínico, en donde se especifica la fisiopatología, factores, causas y diagnóstico de la HPP; La prevención, tratamiento e intervenciones de enfermería y finalmente las Dificultades que se presentaron durante la atención gineco obstétrica.

### Temática 1. Cuadro clínico

#### 1.1. Fisiopatología de la hemorragia postparto

El miometrio es el componente muscular del útero; durante el alumbramiento, sus fibras se contraen y retraen ocasionando engrosamiento y disminución el volumen uterino; la placenta al separarse por sí sola provoca que los vasos sanguíneos se rompan, así produce un sangrado de 600 a 900 ml de sangre, como respuesta se producen contracciones en el miometrio, dando lugar a la formación de un coágulo retroplacentario; esto sucede en aproximadamente 15 a 35 minutos, cuando se extiende el tiempo se considera alumbramiento prolongado. Sin embargo, se puede producir una falla en este proceso fisiológico llamado atonía uterina lo que no permite la contracción y produce la hemorragia^42^^,^^49^.

#### 1.2. Fisiopatología del shock hipovolémico

Debido a la HPP se genera hipoperfusión tisular, lo que ocasiona la pérdida de oxígeno a nivel celular; esto desencadena un estado de shock, caracterizado por trastornos metabólicos intracelulares que culminan en falla orgánica y muerte. Como mecanismo compensador se liberan catecolaminas, por lo cual se activa el sistema nervioso simpático, que a su vez aumenta la frecuencia y contractilidad cardiaca, resistencia vascular sistémica y pulmonar; por consiguiente, disminuye el flujo sanguíneo hacía riñón, bazo, piel y útero, conservando la perfusión cardiaca, cerebral y suprarrenal. Cuando la pérdida de volumen excede el 25% (1500 ml aproximadamente) los mecanismos compensadores fallan, en este momento se genera hipoxia de tejido y en consecuencia acidosis metabólica^44^, ^47^.

#### 1.3. Factores de riesgo de la hemorragia postparto

En varios estudios se ha encontrado que las mujeres pueden presentar condiciones preexistentes y condiciones intraparto, las cuales representan un mayor riesgo de desarrollar HPP. Entre estos los más frecuentes son: parto prolongado, embarazo múltiple, alteraciones placentarias, multiparidad, inducción al parto con oxitocina y polihidramnios^25^^,^^30^^,^^31^^,^^38^^-^^40^^,^^41^^-^^50^, ^51^^-^^55^^,^^58^- ^60^, ^63^.

Ver Tabla 2.

**Tabla 2:** Factores predisponentes asociados a HPP, reportadas en la literatura.

Factores	%
Antecedentes de HPP	4.7%
Multiparidad	53% - 95%
Alteraciones placentarias	95%
Placenta previa	
Retención placentaria	
Placenta acreta	
Desprendimiento placentario	
Edad <20 - >35 años	31.7% - 60%
Corioamnionitis	1.4%
Anemia	6.9% - 34%
IMC> 35kg/m2	24%
Macrosomía fetal	14.8% - 32.9%
Trastornos Hipertensivos	36.1%
Pre eclampsia	
Síndrome de HELLP	
Inducción del parto	28.3% - 84.7%
Embarazo múltiple	61.6% - 95%
Laceraciones del tracto vaginal	35.2%
Desgarros	
Episiotomía	
Polihidramnios	70%
Parto prolongado	100%
Embolia de líquido amniótico	2.8%

#### 1.4. Causas de la hemorragia postparto

Dentro de la literatura se mencionan condiciones clínicas del posparto que son clasificadas como “las 4Ts”: tono, trauma, tejido y trombina que pueden desencadenar complicaciones en esta etapa^1^^,^^25^^,^^29^^,^^31^^,^^36^^,^^53^. Las alteraciones del tono se desarrollan con mayor frecuencia (70%), es característica la atonía uterina presentándose en un 60% - 85% de las mujeres^31^^,^^33^^,^^39^^,^^40^^,^^42^^-^^52^^,^^54^^,^^55^^,^^58^^-^^63^, seguida de la hipotonía uterina (82%)^36^^,^^45^^,^^47^; así como diferentes condiciones placentarias^31^^,^^35^^,^^39^, ^40^^,^^43^^,^^44^^,^^46^^,^^49^^,^^59^^,^^60^^,^^62^, de las cuales en el 81.6% de los casos se presenta la placenta acreta y en un 29%- 54.4% placenta previa, mientras que las restantes son menos comunes: placenta increta 11.8% y placenta percreta 6.6%. Dentro de dichas anomalías las que menos desarrollan HPP son la inversión uterina (2.8% - 5%) y la rotura uterina (2.8%)^35^^,^^39^^,^^40^^,^^43^^-^^46^^,^^54^^,^^55^^,^^62^^,^^63^.

El trauma es la segunda causa de HPP e involucra lesiones del tracto genital como los desgarros y la episiotomía presentándose entre el 19% y 35% de los casos^31^^,^^33^^,^^39^^,^^40^^,^^42^^-^^50^^,^^54^^,^^55^^,^^58^^,^^62^^,^^63^. Por otro lado, ocurre en un 10% - 21% por alteraciones en el tejido, ya sea por anomalías placentarias (9.5% - 36%) o por retención de restos placentarios (9% - 35%)^31^^,^^40^^,^^42^^-^^50^^,^^52^^,^^54^^,^^53^^,^^62^ por último, las causas menos frecuentes son las alteraciones de la coagulación (1% - 7.4%), ya que por lo general se presentan en mujeres con alteraciones hematológicas^31^^,^^35^^,^^39^^,^^42^^-^^46^^,^^48^^-^^50^^,^^55^^,^^62^.

#### 1.5 Diagnóstico de la hemorragia postparto

Según Romero y otros^45^, el personal de enfermería identifica la HPP y activa la respuesta inmediata en el 60% de los casos. Por consiguiente, es pertinente realizar un examen físico exhaustivo, que consiste en la palpación bimanual del útero con el objetivo de identificar ruptura uterina, retención placentaria, coágulos y laceraciones, si se descubren cotiledones incompletos en la placenta se debe limpiar la cavidad uterina^44^^,^^47^; de igual manera se debe inspeccionar el cérvix y la vagina en busca de hematomas o desgarros^44^.

Adicionalmente es indispensable valorar las pérdidas sanguíneas por estimación visual y compresas; la OPS (2018) propone una fórmula para calcular la pérdida de volumen pesando las compresas, teniendo en cuenta que 1 gramo de peso es igual a 1 mililitro de sangre^43^^,^^50^^,^^53^.

*Peso de compresas con sangre (gramos) - Peso calculado de compresas seco (gramos) = Volumen estimado de sangre perdida (mililitros)*

Igualmente se deben obtener la clasificación ABO y Rh para pruebas cruzadas, niveles de he- moglobina, hematocrito y recuento de plaquetas, tiempo de protrombina y tromboplastina en donde se evaluarán alteraciones en la coagulación^44^. Posterior a esto se deben reconocer los signos de inestabilidad hemodinámica y shock los cuales están relacionados con la pérdida de volumen sanguíneo^43^^,^^47^^,^^50^. Ver Tabla 3.

**Tabla 3:** Signos clínicos de HPP.

Pérdida de volumen(%) y ml para una mujer entre 50 - 70 kg	Estado de conciencia	Presión arterial Frecuencia sistólica (mmHg)	Frecuencia **cardiaca (lpm)**	Perfusión	Gasto urinario	Grado de shock
10 - 15% 500 - 1000 ml	Normal	Normal	60-90	Normal	Normal	Compensado
16 -25% 1000 - 1500 ml	Normal y/o agitada	80-90	91-100	Palidez, frialdad	Normal	Leve
26 - 35% 1500 - 2000 ml	Agitada y confusión	70-80	101-120	Palidez, frialdad y sudoración	Oliguria	Moderado
>35% 2000 - 3000 ml	Letárgica, colapso e inconsciente	<70	>120	Palidez, frialdad, sudoración y llenado capilar	Anuria	Severo

*Fuente: Guía de cuidado de enfermería a la mujer en el período posparto. Enfermería basada en la evidencia (EBE). Secretaría Distrital de Salud. Alcaldía Mayor de Bogotá D.C. 2015*

### Temática 2. Cuidados de enfermería

*Prevención de la HPP:* Diferentes autores afirman que se debe capacitar al personal de salud para la utilización de protocolos que impliquen mantener la estabilidad hemodinámica, contar con la experiencia y conocimiento para el manejo de la urgencia obstétrica, identificando los factores de riesgo ^34^^,^^43^^,^^45^^,^^54^^,^^62^.

El manejo activo de la tercera etapa del parto incluye la administración de uterotónicos, la tracción sostenida, el pinzamiento del cordón umbilical asociado con la maniobra de Brandt- Andrews y realizar masaje en el fondo uterino cada 15 minutos durante las primeras 2 horas postparto^31^^,^^39^^-^^43^^,^^45^^-^^49^^,^^51^^-^^53^. El medicamento de elección en la profilaxis es la oxitocina^31^^,^^39^^,^^40^^,^^43^^,^^45^^-^^47^, ^51^^,^^53^, en dosis de 10-30 UI por vía intravenosa (IV) que tienen una duración de acción entre 2-4 horas y 8-12 horas respectivamente y dosis de 5-10 UI vía intramuscular (IM) inmediatamente después del nacimiento. Otras alternativas son misoprostol 400-600 microgramos vía oral o sublingual, lo que reduce el 8% de los casos^31^^,^^39^^,^^46^^,^^52^ o la administración de 100 microgramos de carbetocina^31^^,^^39^ IV o IM; por otra parte, se presenta una reducción eficaz de la HPP con la administración de ácido tranexámico^31^^,^^57^ dentro de las 3 horas posteriores al parto en dosis de 0.5-1 g vía intravenosa.

*Variablesaevaluarenla HPP:* Es indispensable su detección temprana, mediante la monitorización de variables hemodinámicas, se recomienda la toma de los signos vitales cada 15 minutos durante una hora, a excepción de la temperatura^53^^,^^54^. A continuación, se describen de manera fisiológica las variables a monitorizar:

1. *Frecuencia cardiaca.* Como consecuencia de la disminución de la precarga, se presenta una elevación de la frecuencia cardíaca relacionada con la magnitud de la hipovolemia^44^.
2. *Presión arterial.* La pérdida de volumen genera una descarga adrenérgica que aumenta el tono vasomotor, lo que disminuye la presión de pulso y mantiene la presión sistólica. Una vez se ha perdido el 30% del volumen sanguíneo comienza a producirse hipotensión^44^.
3. *Piel.* Para preservar la perfusión cerebral, coronaria y visceral, se activan de mecanismos vasoconstrictores los cuales a su vez son los responsables de producir signos como piel fría, pálida, sudorosa y llenado capilar prolongado^44^.
4. *Diuresis.* Al presentarse una depleción de volumen se genera una redistribución del flujo sanguíneo hacia otros órganos vitales lo que provoca oliguria^44^.
5. *Acidosis metabólica. S*i el shock progresa se activa la ruta metabólica anaeróbica la cual produce ácido láctico, mientras que la hipoperfusión tisular hepática disminuye el lactato, lo que conlleva a acidosis metabólica^44^.
6. *Alteraciones del laboratorio.* En caso de hipovolemia se conserva agua y sodio a nivel renal, esto produce aumento de nitrógeno ureico sanguíneo, relación BUN/creatinina >20, osmolaridad urinaria >450 mmol/kg y sodio urinario <25 mEq/l^44^.
7. *Fibrinógeno.* Es fundamental para la agregación plaquetaria, sin embargo, en la HPP sus niveles descienden y pueden indicar la necesidad de realizar transfusión masiva de hemoderivados, así como riesgo de desencadenar coagulopatía^26^^,^^47^.
8. *Lactato.* Es crucial para determinar si se necesita una transfusión masiva de hemoderivados, puesto que el lactato es un producto celular del metabolismo anaerobio^26^^,^^47^.
9. *Índice de shock (IC).* Parámetro clínico que indica la pérdida del volumen sanguíneo durante el postparto, la necesidad de transfusión masiva y refleja el estado hemodinámico de la paciente. El índice de shock es calculado con la fórmula: (Frecuencia cardíaca materna ÷ Presión arterial sistólica = Si IC mayor o igual a 0.9: transfusión masiva) ^24^^-^^27^^,^^41^^,^^44^^,^^51^^,^^53^^,^^60^.

Los principales Diagnósticos relacionados se describen a continuación con las intervenciones recomendadas. Ver Tabla 4.

**Tabla 4:** Diagnósticos de enfermería.

Diagnósticos de enfermería	NIC
Déficit de volumen de Líquidos R/C Pérdida activa del volumen de líquidos E/P Disminución de la diuresis, disminución de la presión arterial, membranas mucosas secas, sed, debilidad.	Realizar sondaje vesical. Monitorizar estado hemodinámico cada 15 minutos. Administrar terapia IV soluciones cristaloides, preferiblemente tibias (salina normal, lactato de Ringer), coloides, hemoderivados, oxitócicos o inotrópicos según prescripción.
Déficit de líquidos R/C pérdida de sangreexcesiva secundario a atonía uterina. Disminución del gasto cardiaco	Realizar control de líquidos administrados y eliminados. Mantener una vía endovenosa permeable.
R/C alteración de la precarga E/P piel fría y sudorosa, disminución de los pulsos periféricos, prolongación del tiempo de llenado capilar.	Observar los niveles de hemoglobina, hematocrito, TP, TTP, fibrinógeno, recuento de plaquetas. Mantener la posición adecuada que asegure la perfusión periférica con las piernas elevadas. Aumentar la frecuencia de masaje en el fondo uterino
Riesgo de shock R/C hipovolemia ^1^^) (^^44^^,^^49^^,^^54^^,^^63^ Hipotermia^54^.	Cuantificar la sangre perdida Auscultar los sonidos pulmonares y verificar si hay crepitantes u otros sonidos.
Deterioro del intercambio de gases R/C desequilibrio en la ventilación - perfusión E/P pH arterial anormal, patrón respiratorio anormal, somnolencia, taquicardia^44^.	Monitorizar los niveles de electrolitos^40^, ^42^, ^44^, ^49^, ^53^, ^54^, ^60^, ^61^, ^63^. Observar el color y la temperatura de la piel. Ajustar la temperatura ambiental a las necesidades del paciente. Administrar oxigenoterapia según corresponda^44^.

*Tratamiento farmacológico:*

Las intervenciones farmacológicas van dirigidas hacia el control inmediato de la pérdida de volumen sanguíneo, con la finalidad de mitigar el riesgo de shock hipovolémico. Por otro lado, los uterotónicos^28^ son usados ya que aumentan la contractilidad y el tono uterino; los fármacos de primera elección son la oxitocina y la ergometrina^24^, puesto que su inicio de acción es de 2 a 3 minutos, el primero maneja una dosis inicial de 10 UI IM o 10-40 UI IV y una dosis máxima de 60 UI/día; el segundo tiene una dosis inicial de 0.2-0.4mg IV o IM en 15 a 20 minutos, cada 4 a 6 horas hasta un máximo de 1 mg; como alternativa se administra misoprostol 600-1000 mcg sublingual o rectal; carboprost 0.25 mg IM cada 15 a 90 minutos en un máximo de 8 dosis y ácido tranexámico 1g IV cada 4 horas, máximo 4 g^32^^,^^51^.

Las dosis de mantenimiento deben continuar simultáneamente: 10-30 UI IV de oxitocina o 100 µg de bolo IV de carbetocina^31^. estos fármacos deben estar sellados, lejos de la luz y mantener a temperatura de hasta 30ºC^1^^,^^39^^,^^44^^,^^45^^,^^47^^,^^48^^,^^53^^,^^54^.

*Tratamiento no farmacológico:*

En la literatura se listan diferentes alternativas, tomando como primera medida la reposición de líquidos cristaloides y/o hemoderivados según las pérdidas^24^^-^^27^^,^^31^^,^^34^^,^^36^^,^^37^^,^^42^^-^^47^^,^^49^^,^^51^^,^^55^^,^^59^^,^^61^^,^^63^. Se recomienda la administración de plasma fresco congelado y/o glóbulos rojos; se debe canalizar una vía periférica con un catéter de gran calibre (16-18)^29^^,^^42^^,^^43^ de uso exclusivo para la transfusión de hemocomponentes, en caso de emergencia se administran 2 unidades de glóbulos rojos O-1, ^43^^-^^45^. Se sugiere 12-15 ml/kg de plasma fresco congelado por cada 6 unidades de glóbulos rojos, mantener la monitorización de las constantes vitales, orientar y vigilar las reacciones adversas presentes durante la transfusión1,^31^^,^^37^^,^^43^.

Por otro lado, controlar la diuresis a través de una sonda Foley; el vaciado vesical favorece la contracción uterina, el volumen diurético adecuado es de > 30 ml/hora^29^^,^^32^^,^^42^^,^^63^. En relación a otras alternativas se encuentra el balón Bakri, es un dispositivo de silicón mínimamente invasivo que se utiliza en el taponamiento uterino, tiene doble luz para vigilar y cuantificar el sangrado uterino, aumenta la presión del parénquima y la vasculatura uterina; debe llenarse con solución salina hasta producir suficiente taponamiento. Este método ha demostrado una eficacia del 90% durante 12-24 horas^25^^,^^31^^,^^36^^,^^42^^,^^44^^,^^47^^,^^48^^,^^50^^,^^51^^,^^53^^,^^55^^,^^59^^,^^60^. De otra manera, se realiza compresión manual con gasas estériles y compresas en la cavidad uterina; tiene una efectividad de 2 a 3 horas aproximadamente, y/o compresión bimanual uterina en donde se coloca un puño a través del tracto vaginal y con la otra mano se comprime el fondo uterino^39^^,^^42^^,^^48^^,^^50^.

Finalmente, el traje anti choque es una prenda de seis segmentos (maléolos, piernas, muslos, pelvis y abdomen) que ejercen presión de 20 a 40 mmHg para disminuir la irrigación sanguínea en estas regiones para favorecer el flujo de sangre a órganos vitales, contrarrestando el shock hipovolémico en un tiempo estimado de 48 a 72 horas^25^^,^^28^^,^^53^^,^^61^.

Recomendaciones de uso: 1. Colocar a la paciente sobre el traje abierto. 2. Verificar la posición: el segmento superior debe quedar inmediatamente debajo de la última costilla y el balón de presión sobre el ombligo. 3. Cerrar el traje iniciando por los segmentos de los tobillos y ascender. 4. Verificar que el ajuste colocando uno o dos dedos debajo del borde superior del segmento. 5. Preguntar si puede respirar. 6. Vigilar la aparición de disnea o la disminución del gasto urinario como signos de que el traje está demasiado ajustado^29^.

Para retirarlo se debe realizar desde el punto distal hasta el proximal, aplicando la regla de los 20/20, consiste en que antes de pasar de un nivel a otro deben transcurrir 20 minutos, en este lapso se debe verificar que la presión arterial sistólica no descienda más de 20 mmHg o la frecuencia cardiaca aumente más de 20 latidos por minuto. No debe retirarse súbitamente puesto que produciría una redistribución del volumen sanguíneo hacia los miembros inferiores, aumentando la probabilidad de un colapso vascular súbito^29^^,^^61^.

*Tratamiento del shock hipovolémico:*

Durante el manejo del shock hipovolémico se realiza una resucitación sistémica; en primer lugar, se asegura la vía aérea a través de intubación endotraqueal y se verifica el riesgo de aspiración. En segundo lugar, se canalizan 2 accesos venosos de gran calibre para administración de líquidos IV, se aporta 1-2 litros de volumen durante la primera hora, con vigilancia estricta de los signos vitales; las soluciones utilizadas son los cristaloides, coloides y sangre^44^. Por último, se administran fármacos vasopresores como Desmopresina 0.3µg/kg, uterotónicos como Oxitocina 30 UI/30 min y si estos medicamentos son ineficaces, se administra Sulprostone 500 mg en 1 hora; y adicional Bicarbonato de sodio para contrarrestar la acidosis láctica^31^^,^^32^.

### Temática 3. Dificultades en la atención obstétrica

Se identifican barreras que impiden que se brinde una atención oportuna y de calidad aumen- tando la incidencia de eventos como HPP, la detección y tratamiento apropiado permite evitar complicaciones como el shock hipovolémico y la muerte^34^. En los países de recursos bajos y medios^62^ existe una mayor incidencia de HPP debido a factores como: atención prenatal inadecuada^34^^,^^39^; la autonomía de la paciente de no seguir un control prenatal ya sea por preferencias culturales, atención de baja calidad, acceso limitado a los servicios de salud o condición económica^42^^,^^48^^,^^51^; atención en hospitales de primer nivel con recursos limitados para brindar un tratamiento adecuado^59^^,^^60^ y demora en el traslado a instituciones de tercer o cuarto nivel para la atención de situaciones de complejidad presentadas^24^^,^^40^^,^^60^.

Adicional a esto, la falta de conocimientos y habilidades en los profesionales de enfermería^38^^,^^42^^,^^45^^,^^48^ contribuye a una identificación inoportuna de los signos de HPP^24^^,^^36^ lo que conlleva a un diagnóstico tardío^40^, por consiguiente, el retraso de las intervenciones^34^^,^^40^^,^^48^. Todas estas situaciones representan contrariedad en la atención a las pacientes^51^.

Asimismo, en las instituciones existe baja adherencia a las guías de práctica clínica para activar el código rojo^36^^,^^42^^,^^48^ e intervenir ágilmente con el equipo interdisciplinario según los requerimientos. Por consiguiente, se recomienda a los profesionales de enfermería desarrollar protocolos que aborden las acciones de cuidado^62^, y potenciar sus conocimientos sobre la HPP, tratamiento farmacológico, uso de equipos, habilidad para realizar procedimientos^45^; además, de establecer canales de comunicación con los familiares sobre la condición de la paciente, con el fin de mejorar y fortalecer el rol como profesionales^34^^,^^42^^,^^43^.

## Discusión

La HPP es el principal factor de riesgo de la mortalidad materna siendo este un indicador de desarrollo de los países, por lo tanto es indispensable que se propenda por evitar al máximo estas complicaciones en las mujeres gestantes, para esto el profesional de enfermería debe asumir acciones frente a la seguridad de la paciente, desde la evaluación y análisis de los casos de mortalidad, la capacidad resolutiva de las instituciones en salud para identificar errores y/o barreras en la atención, promoción del trabajo en equipo, fortalecimiento de competencias para la comunicación e implementación guías de práctica clínica basadas en la evidencia^64^.

Es imprescindible que el profesional de enfermería establezca medidas de prevención universales que permitan reconocer los signos, síntomas, factores de riesgo e impacto psicológico, y de igual manera perciba que la HPP genera dolor, miedo y angustia a la mujer; por tal motivo, su intervención debe enfocarse en brindar cuidados de calidad que permitan estabilizar estos aspectos, con el fin de que la madre se encuentre en la capacidad de cumplir eficazmente su rol maternal, segura y confiada de que el equipo de enfermería tomará decisiones basadas en el respeto conservando la intimidad del binomio^65^^,^^66^.

Adicional a esto, se resalta la importancia de la contribución del lenguaje estandarizado en las taxonomías NANDA, NIC y NOC donde se relacionan los dominios, patrones y necesidades con los procesos de atención de enfermería, teniendo en cuenta las intervenciones prioritarias en las situaciones de riesgo para las pacientes, disminuyendo así indicadores de morbimortalidad materna^67^.

A partir del tratamiento farmacológico, se recomienda el uso de uterotónicos como la oxitocina la cual es esencial para contrarrestar la HPP y por consiguiente el shock hipovolémico; su uso varía de acuerdo a la disponibilidad y grado de la hemorragia. Según la literatura, el uso concomitante de Misoprostol con oxitocina o la administración únicamente de Carbetocina son más efectivas para prevenir sangrado ≥500 ml, y la combinación de Ergometrina con oxitocina para prevenir HPP ≥1000 ml^68^.

Según la evidencia, se prefiere el uso del balón Bakri ya que es asequible por su bajo costo; teniendo cuenta que es un dispositivo de primera línea en el manejo avanzado no quirúrgico y no farmacológico de la HPP, que debe emplearse si los uterotónicos de elección fallan. Si este no resulta efectivo para el control de la hemorragia se confirma la necesidad de laparotomía u otra intervención quirúrgica^69^.

## Conclusiones

Es pertinente realizar un examen físico exhaustivo, que consiste en la palpación bimanual del útero con el objetivo de identificar y reconocer los factores de riesgo, causas (4 Ts), signos de inestabilidad hemodinámica, y de shock hipovolémico a través de la evaluación continua de los criterios de severidad para prevenir la HPP hasta en un 93%.

Respecto a los diagnósticos de enfermería relacionados directamente con la HPP, es de relevancia destacar que están orientados a la pérdida de volumen de líquidos y la inestabilidad hemo- dinámica que conlleva al deterioro de intercambio de gases, la hipotermia y disminución del gasto cardíaco; para lo cual las intervenciones planteadas en la taxonomía (NIC) se enfocan en brindar cuidados de calidad, todo esto con el fin de realizar un manejo y tratamiento oportuno para evitar complicaciones como la muerte.

Finalmente, se evidencia que la HPP es la segunda causa de mortalidad materna con mayor incidencia en países de recursos bajos y medios debido a dificultades en la atención obstétrica. Por tanto, se hace necesario capacitar a los profesionales de enfermería fomentando la adherencia de guías de práctica clínica, así como la implementación de medidas terapéuticas y/o farmacológicas; además de contar con la experiencia y conocimiento para el manejo de la HPP.
